# An open-source solution for advanced imaging flow cytometry data analysis using machine learning

**DOI:** 10.1016/j.ymeth.2016.08.018

**Published:** 2017-01-01

**Authors:** Holger Hennig, Paul Rees, Thomas Blasi, Lee Kamentsky, Jane Hung, David Dao, Anne E. Carpenter, Andrew Filby

**Affiliations:** a Imaging Platform at the Broad Institute of Harvard and MIT, 415 Main St, Cambridge, MA 02142, USA; b Dept. of Systems Biology & Bioinformatics, University of Rostock, 18051 Rostock, Germany; c College of Engineering, Swansea University, Singleton Park, Swansea SA2 8PP, UK; d Helmholtz Zentrum München — German Research Center for Environmental Health, Institute of Computational Biology, 85764 Neuherberg, Germany; e Flow Cytometry Core Facility, Faculty of Medical Sciences, Newcastle University, Newcastle upon Tyne, NE1 7RU, UK

**Keywords:** IFC, imaging flow cytometry, Imaging flow cytometry, Machine learning, Open-source software, High-throughput, Feature selection, Profiling

## Abstract

Imaging flow cytometry (IFC) enables the high throughput collection of morphological and spatial information from hundreds of thousands of single cells. This high content, information rich image data can in theory resolve important biological differences among complex, often heterogeneous biological samples. However, data analysis is often performed in a highly manual and subjective manner using very limited image analysis techniques in combination with conventional flow cytometry gating strategies. This approach is not scalable to the hundreds of available image-based features per cell and thus makes use of only a fraction of the spatial and morphometric information. As a result, the quality, reproducibility and rigour of results are limited by the skill, experience and ingenuity of the data analyst. Here, we describe a pipeline using open-source software that leverages the rich information in digital imagery using machine learning algorithms. Compensated and corrected raw image files (.rif) data files from an imaging flow cytometer (the proprietary .cif file format) are imported into the open-source software CellProfiler, where an image processing pipeline identifies cells and subcellular compartments allowing hundreds of morphological features to be measured. This high-dimensional data can then be analysed using cutting-edge machine learning and clustering approaches using “user-friendly” platforms such as CellProfiler Analyst. Researchers can train an automated cell classifier to recognize different cell types, cell cycle phases, drug treatment/control conditions, etc., using supervised machine learning. This workflow should enable the scientific community to leverage the full analytical power of IFC-derived data sets. It will help to reveal otherwise unappreciated populations of cells based on features that may be hidden to the human eye that include subtle measured differences in label free detection channels such as bright-field and dark-field imagery.

## Highlights

• Imaging flow cytometry enables potentially powerful, multiplexed single-cell analysis.
• Data analysis techniques for imaging flow cytometry are largely manual and subjective.
• Our machine learning workflow identifies phenotypes in imaging flow cytometry.
• The workflow uses open-source software and does not require computational expertise.

## 1 Introduction

It is now widely accepted that cellular and molecular heterogeneity pervades all biological systems [1], [2]. This creates a complex set of challenges for understanding how individual cells within heterogeneous communities interact with one another in order to determine the phenotype and function of higher organisms with respect to both healthy and disease states. Our ability to appreciate biological heterogeneity is limited by the resolving power of the analytical approaches at our disposal. At the methodological level, there is currently a massive paradigm shift away from so called “bulk” analysis techniques toward single cell-focused approaches that are able to cope far better with the challenges posed by heterogeneity [3]. “Cytometry” translates in literal terms to mean “cell measurement” and can best be described as the derivation of numbers from the measurement of large populations of single cells. While extremely powerful, it is a significant challenge to derive meaningful, objective conclusions from the high parameter output inherent to nearly all cytometric approaches. While cytometric technologies such as fluorescence-based flow and mass cytometry can currently measure 30–40 parameters per cell [4], the parameter output from image-based cytometry systems can be almost infinite and often continuous (non-discrete) in nature.

**Fig. 1:**
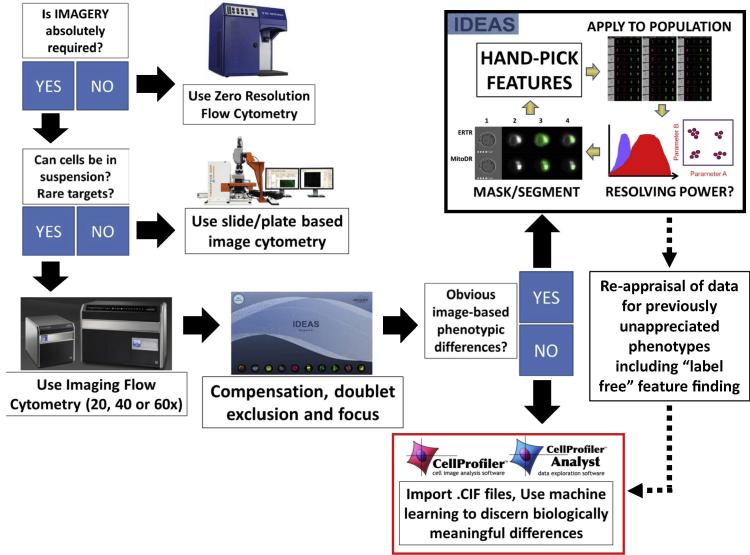
Guidance on choosing cytometric method and analysis method. Any researcher who wants to use cytometry technology to ask a defined question should consider “what is the best approach” based on the question. For example if morphological/spatial information is not required then so-called “zero resolution flow cytometry” is best. If however the question absolutely requires imagery, then the sample type should next be considered, is it tissue? Can it be disaggregated? Could it be analysed in such a way that the spatial relationship of individual cells is lost? In our experience, IFC is best applied to situations where the cells biology can still be analysed when in suspension. This could still be disaggregated tissue or adherent cells and not just cells that exist in suspension. If the target cell population is rare, then suspension-based high throughput analysis is often necessary to collect sufficient events for statistical confidence. Once the IFC data is collected, several options can be chosen for data analysis. This figure summarises these options in light of our proposed solution. The historical option is to rely entirely on IDEAS software to perform a potentially subjective, iterative image analysis that involves adapting the masking/segmentation rules to best identify key pixels within an image channel and then to try and select the best feature calculated on these pixels with the aim of resolving different phenotypes from one another. This approach can be partially automated using the so-called “find the best feature” method. We propose however that a deeper analysis of features is more appropriate to IFC data sets. In this regard we have developed and validated a machine learning-based approach to analyse IFC data that has been corrected and compensated in IDEAS (.rif to .cif conversion). We then use the open source image analysis platforms CellProfiler and CellProfiler Analyst to better interrogate the imagery. Even in cases where the IDEAS-based iterative approach works very well, as is often the case when the outcome is well defined, there may be benefit to re-analysing these data using the approach presented here. It may uncover unappreciated features - in our own experience, this allowed us to perform a label-free classification of cell cycle stages, thus eliminating the need to add potentially confounding dyes to our cells [6].

One very powerful image-based cytometric technology is imaging flow cytometry (IFC, Fig. 1). It combines the high-throughput, multi-parameter capabilities of conventional flow cytometry with the current capability to capture up to 12 spatially registered multi-spectral images for each cell as it passes through the system [5]. These imaging channels can capture label-dependent fluorescence signals (currently up to 10) as well as transmitted bright-field and laser side scatter (dark-field) information, the latter of which do not require any introduced fluorescence (label-free). IFC very much fits the paradigm of “image cytometry” as it produces quantifiable image data in a high throughput, multi-parameter format. This ensures that a fair, unbiased comparison can be made between the output images so that any measured differences can be considered biological rather than an artefact of variable imaging conditions.

In many cases the unique capabilities of IFC to deliver high-throughput, multispectral, spatially registered imagery has been essential to the development of new assays to ask novel, cutting edge biological questions. Most IFC-based assays take advantage of the technologies’ inherent ability to measure fluorescence signals with spatial context. Such assays include measuring nuclear translocation [7], mitochondrial localisation [8], co-localization assays using “similarity” features [9], calcium signalling at the organelle level [10], organelle inheritance during mitosis [11], cell cycle phases [12], receptor activity [13], asymmetric cell division [14], [15], [16], fission yeast cell cycle [17], dendritic cell morphology [18], autophagy [19], [20], detection of DNA damage foci [21], [22] and modelling intracellular infection [23]. Most, if not all, of these assays would not be possible using traditional flow cytometry (lack of spatial information) or conventional imaging techniques (low throughput and poor quantitation).

Despite IFC being available to the research community for over 10 years it is still often referred to as a “new and emerging technology”. In reality this is no longer the case. While new applications of the technology continue to be developed on a regular basis, the data analysis methods for IFC have noticeably lagged behind, and certainly fall significantly short of their potential. In fact, it could be argued that data analysis presents the single biggest bottleneck/barrier to a more comprehensive adoption of IFC by the research and clinical diagnostic communities.

The most common approach to IFC data analysis is to use the proprietary analysis software called IDEAS (manufacturer supplied). This software is extremely powerful, allowing the user to explore a range of image features derived from each individual cell. There are a number of pre-calculated features that measure pixel-based parameters (including morphological, intensiometric and texture based features) using default cell segmentation algorithms that automatically generate masks for each available imaging channels on a per cell/object basis. It is also possible within the IDEAS software to derive novel user-defined features based on either the default channel masks or completely novel masks/segmentations that can be constructed using a powerful suite of adaptation algorithms. Briefly, the latter allows the user to adapt the default channel masks using a set of predefined rules to hone the segmentation in such a way as to ensure the mask is optimised with respect to what pixels it identifies within the image frame as being “of interest” for subsequent analysis. New features can then be calculated from within the masked area to hopefully better resolve subtle biological differences in populations/treatment groups.

The majority of the innovative applications mentioned previously have used this manual, iterative IDEAS-based approach. However, this approach can sometimes lead to unconvincing conclusions and poor reproducibility of results because there are literally thousands of strategies that could be employed for data analysis that can yield conflicting conclusions. A significant contributor to this variation is the intimate relationship between how well a given mask is able to identify the right groups of pixels in an image and the resolving power of any feature derived from the pixel properties under that mask [24]. For example, a logical feature could be selected but the resolving power will be compromised by poor pixel masking. A good example of this is using the “spot count” feature to resolve cells in anaphase from those in metaphase. If the default nuclear dye channel mask is used, it tends to be very permissive and will mask both nuclear poles in an anaphase cell as a single entity. In this case calculating the “spot count” of this mask will not resolve metaphase from anaphase (both will give a value of 1). However if we adapt the stringency of the default nuclear channel mask so that it now identifies two separate foci, the spot count will be a powerful resolving feature [24].

Recently an analysis method with some aspects of machine learning has been proposed, known as the “find the best feature” approach [19]. It involves manually selecting around 20–30 cells that exemplify each of the user-observed phenotypes within the sample (including controls), possibly requiring some new basic masks to be constructed within the IDEAS software. IDEAS is then used to calculate a range of features from these masks. The resolving power of each individual feature associated with a specific channel masks is then ranked using the Fisher discrimination ratio (Rd) to provide a numerical value for the degree of statistical separation between two potentially overlapping distributions. In this case the two distributions are derived from the hand-tagged populations, one containing cells that exemplify a given observed phenotype and the other containing cells that do not. The feature/mask combination with the highest Rd value is then selected and applied to the entire data file to assess the resolving potential for larger numbers of cells. There are numerous potential problems with this approach, not least the fact that selecting so few cells to test the resolving power of a feature may confound the ranking due to effects of under sampling. For example, if we were to repeatedly sample 20–30 cells from a population of 4000 cells and “find the best feature”, it is possible that each time we will get a totally different top ranking feature due to effects of “under sampling”. In reality we should test the resolving power of a set of features using between 100 and 500 cells in order to reach the point of “diminishing returns”. Using a manual approach, this would be very time consuming.

By comparison, the field of image-based profiling is much more advanced: hundreds of morphological features are extracted from each cellular image acquired by conventional microscopy, and the “profiles” of these cells are compared and/or subjected to machine learning to identify biologically important differences among them [25], [26]. Until now, this approach has been hard to employ for IFC-derived data because of the challenges around the proprietary file formats and a workflow that is often highly subjective, relying far too much on individual human input.

We previously developed a prototype workflow in our effort to develop a label-free assay for cell cycle analysis, using machine learning on the bright-field and dark-field images image setts from an imaging flow cytometer [6]. That analysis consisted of several steps (see Methods section for details) that required the use of commercial software (MATLAB) both before and after using the open source software CellProfiler. In addition, significant expertise in computer programming and machine learning was required to follow the protocol. The pipeline also required the IDEAS software to extract a single tiff image per fluorescence channel for each cell, which for even modest cell counts generated very large numbers of individual images, typically in the hundreds of thousands for high-throughput studies, causing significant problems for file systems, file handling and processing.

Here we describe development of a fully open-source software workflow to enable user-friendly machine learning based analysis of imaging flow cytometry data. The workflow is a step towards “systems cytometry”, i.e. a systems approach to the quantitative image analysis of single cells in high-throughput using advanced data analysis methods.

## 2 Material and methods

### 2.1 Data acquisition in imaging flow cytometry

We briefly review data acquisition in imaging flow cytometers, for a more extensive overview see [27]. Fluorescently labelled or unlabelled cells in solution are run through the *ImageStream* or *FlowSight* (Amnis) imaging flow cytometer and the data is acquired using the INSPIRE control software. Much like traditional flow cytometry, appropriately stained cells should also be measured as controls in order to perform compensation before any analysis is carried out. The INSPIRE acquisition software generates data in the form of a raw image file (.rif file) which can then be directly loaded into IDEAS for further analysis. When the .rif file is loaded into IDEAS, a compensation matrix generated from the fluorescence control experiments can be used to produce a compensated image file (.cif file). In the IDEAS environment, the user can plot features derived from the bright-field, dark-field and fluorescence single cell images in the form of histograms or bivariate scatter plots. Gating can be performed using these plots to generate sub-populations that can be then be studied in further detail. The plots, gating and sub-population information from a session can then be saved as a data analysis file (.daf file). It is also possible to generate individual tiff images from each channel for each cell to analyse outside of the IDEAS framework.

**Fig. 2:**
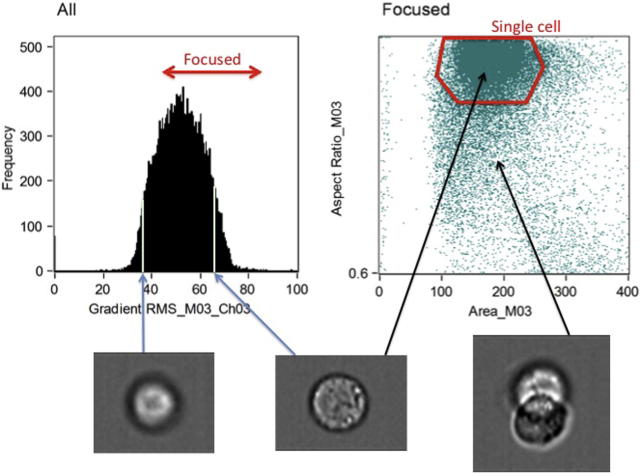
In-focus single cells are gated from the population using bright-field images. Left: cells with a sufficiently high gradient RMS are in-focus (left). Right: objects with a high aspect ratio (a measure of circularity, y-axis) and a mask area that is neither too high nor too low (x-axis) represent single cells.

IDEAS is especially suited for visually inspecting the data irrespective of the further analysis pipeline the user wishes to perform. The important first steps of identifying out-of-focus cells and removing debris or multiple cells are best carried out using this software platform. IDEAS suggests using a measure of the gradient RMS of the bright-field image to determine the focus quality of each cell. By gating the high values in the gradient RMS histogram a subpopulation of in-focus cells is defined (Fig. 2, left). The next step is to identify the single cells by plotting the cell mask aspect ratio versus the cell mask area. A 2D gating window is defined to select cells with an aspect ratio close to 1, which removes clumped cells, while also rejecting high and low areas, which removes debris (Fig. 2, right). Once subpopulations are identified via gating they can be saved as a new .cif file in IDEAS, which serves as the starting point for our protocol.

### 2.2 From data acquisition to high-throughput data analysis

To enable the application of advanced high-throughput data analysis to imaging flow cytometry, we developed a new protocol to harvest and analyse the rich information in images acquired via imaging flow cytometers. Our aim is to provide an open-source protocol that enables user-friendly data processing and extraction of hundreds of features in high-throughput and connects to state-of-the-art data analysis based on machine learning techniques. As discussed above we previously developed a methodology for using high throughput data analysis techniques on imaging flow cytometry data; however, the pipeline required significant computational skills and bespoke MATLAB scripts.

**Fig. 3:**
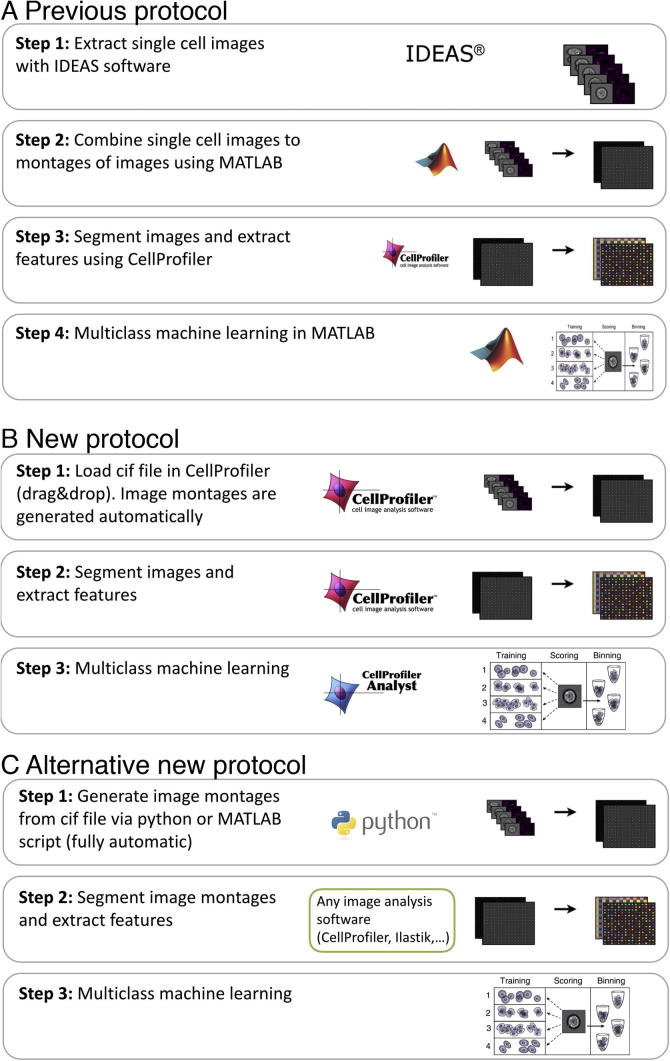
(A) Previous protocol for high-throughput data analysis for imaging flow cytometry [6]. (B) New protocol for high-throughput data analysis in imaging flow cytometry, built from open-source, user-friendly software. (C) Alternative new protocol for high-throughput data analysis in imaging flow cytometry, describing use of various alternate tools at each step.

Our previous protocol consists of the following steps (Fig. 3A).

1. Extract hundreds of thousands to millions of single cell images (tif files) from a single .cif file using IDEAS software and store them to disk as individual files.
2. Pre-process the single cell images: Combine single cell images to montages of 15 × 15 images using a MATLAB script.
3. Segment images and extract hundreds of features per cell per channel, e.g., using CellProfiler. A table of features for each cell can then be exported in a variety of different formats e.g. csv, mat.
4. Downstream data analysis (such as machine learning, feature selection, data visualization, etc.) can then be applied using bespoke code to enable data analysis in high-throughput. In our protocol, we provided MATLAB scripts for this step.

While this protocol was successful in allowing the application of advanced multivariate techniques on imaging cytometry data, several issues limit its application. Many IFC analysts find working with MATLAB scripts to be outside their skill level and thus require computational assistance. Also, handling the hundreds of thousands up to millions of individual tiff images for each channel for each cell files is very slow and difficult to manage for the computer’s file system. We have experienced problems exporting, moving or deleting such a high number of files causing the system to slow down, crash and fragment the disk drive.

To minimise the complexity of the pipeline, we developed an improved protocol that makes imaging flow cytometry data analysis in high-throughput much more streamlined and user-friendly. One major advance is to keep the individual cell tiffs within the cif file container and modify CellProfiler to allow the input of the cif file via the ‘drag and drop’ interface. This eliminates the prior first step, extracting hundreds of thousands or millions of single tiff images and creating tiles therefrom.

The main reason for tiling the single cell images (besides not obstructing the file system) is computational speed: CellProfiler operates image-wise and can quickly detect hundreds or thousands of objects in an image. Linearly crawling through a million tiny images is not practical or feasible in a reasonable time given the overhead of opening individual image files. Instead, the new protocol handles about 1000 tiled images (with 1000 cells in each image tile) when analysing a million cells.

We access the cif file with a new cif file reader, which we contributed to Bio-Formats (http://loci.wisc.edu/software/bio-formats). Bio-Formats is a community driven project with a standardised application interface that supports open source analysis programs like ImageJ, CellProfiler and Icy, informatics solutions like OMERO and the JCB DataViewer, and commercial programs like MATLAB. As such, a cif file can now simply be loaded by those programs. We have integrated the cif file reader into the ‘Images’ module in CellProfiler, via BioFormats, allowing the direct input of the individual cell images for all channels (bright-field, dark-field and fluorescence channels). We have also implemented the tiling of the single cell images within CellProfiler, which removes the need for the software solution in step 2 of our previous protocol. Our new imaging flow cytometry protocol is as follows (Fig. 3B, using CellProfiler):

1. Load .cif file in CellProfiler (drag & drop).
2. Segment images and extract hundreds of features per cell per channel using CellProfiler. An example pipeline can be found at http://cellprofiler.org/imagingflowcytometry/index.html.
3. Multiclass machine learning using CellProfiler Analyst.

In addition, the protocol has also become more streamlined if alternate image analysis or data mining software is preferred (Fig. 3C):

1. Run a Python or MATLAB script to automatically generate tiles of ∼1000 single cell images per tile. A script for this step is available on the website http://cellprofiler.org/imagingflowcytometry/index.html.
2. Load image tiles in your preferred image analysis software such as ilastik, CellProfiler, etc. and analyse images. Export features as .csv file.
3. Multiclass machine learning using any programming language, data analysis tool or visualization tool.

In ([6]), we reported the label-free classification of the cell cycle phases using supervised machine learning techniques on bright-field and dark-field images only. Such high-throughput analyses of IFC data can now be streamlined in a smooth and user-friendly way, making machine learning techniques more accessible.

Supervised machine learning is a powerful approach, where the computer “learns” to recognize cells meeting certain criteria, based on examples provided by the biologist expert. It relies on the hundreds of morphological parameters that have been measured for each cell.

Although any programming language, data analysis tool or visualization tool can be used based on the extracted features, CellProfiler Analyst is a particularly user-friendly option we tested in our protocol. CellProfiler Analyst is free open-source software for exploring and analysing large, high-dimensional image-derived data. It includes machine learning tools for identifying complex and subtle phenotypes [28]. CellProfiler Analyst has recently been updated to include multi-class classification, and it now offers a variety of supervised machine learning techniques [29].

### 2.3 Example: label-free cell-cycle classification of Jurkat cells

We demonstrate our new protocol of analysing IFC data in high-throughput by predicting the cell cycle phase of Jurkat cells based on bright-field and dark-field images only. While the cell-cycle phase can be determined using fluorescent markers of various stages of mitosis, we previously showed that by extracting hundreds of features and using machine learning techniques, it is possible to accurately predict the cell cycle phase without the use of any markers [6]. This method facilitates non-destructive monitoring of cells, avoiding potentially confounding effects of fluorescent stains while maximizing available fluorescence channels.

We will consider the following 5 classes: interphase, and the 4 mitotic phases: prophase, metaphase, anaphase and telophase. The raw images stem from an ImageStream platform where 32,255 asynchronously growing Jurkat cells have been imaged. We use the same Jurkat cell data set from [6] in order to demonstrate the individual steps of the new protocol and to compare our results with a previous benchmark. As controls, the cells were fixed and stained with PI (propidium iodide) to quantify DNA content and a MPM2 (mitotic protein monoclonal #2) antibody to identify mitotic cells. These fluorescent markers were used to annotate the cells with the ground truth (expected results) needed to train the machine-learning algorithms and to evaluate the predictive accuracy of our label-free approach. The ground truth was obtained through gating in IDEAS by using the features from the fluorescent marker channels.

Step 1: Image montages are generated from a .cif file via an automated python script. The script directly reads the .cif file and writes the image montages to disk within seconds (download the script from http://cellprofiler.org/imagingflowcytometry/index.html).

Step 2: We load the montages into CellProfiler (drag & drop) and run a pipeline to measure hundreds of features in bright-field and dark-field (download the pipeline from http://cellprofiler.org/imagingflowcytometry/index.html). The pipeline exports the measurements as a csv file, which can be used with any programming language for downstream data analysis such as machine learning. In addition, the pipeline exports a CellProfiler Analyst properties file (with an SQLite database file). The properties file is a simple text file that can be edited with any text editor. The properties file includes a section where features can be excluded from the classifier; in our case we exclude irrelevant features such as location or angular orientation of the cells (download a sample properties file from http://cellprofiler.org/imagingflowcytometry/index.html).

**Fig. 4:**
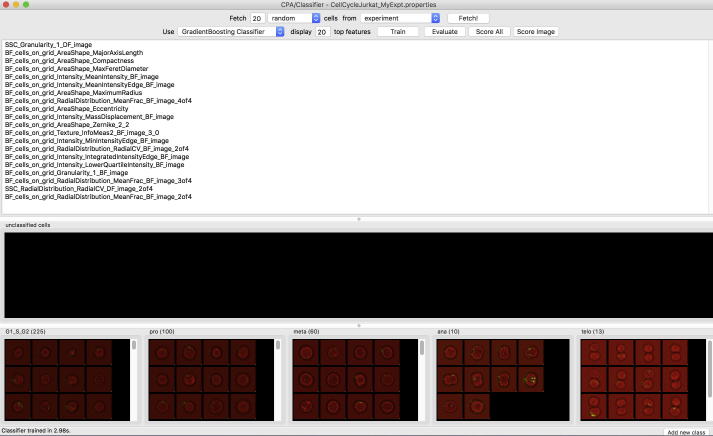
Classification of the cell cycle of Jurkat cells using machine learning in CellProfiler Analyst. The cell images can be sorted via drag & drop into the five different bins at the bottom, which are interphase (G1/S/G2) and the four mitotic phases: prophase (pro), metaphase (meta), anaphase (ana) and telophase (telo). The classifier, here GradientBoosting, is first trained (train button) and then the training set is cross-validated (evaluate button). With the score all button one can predict the cell cycle phase of all cells in the data set.

Step 3: Load properties file into CellProfiler Analyst for the machine learning. See Fig. 4 for details [30], and the online manual for an introduction to machine learning using CellProfiler Analyst (http://cellprofiler.org/CPA). CellProfiler Analyst now includes several machine learning algorithm options; for our purposes, we chose a GradientBoosting classifier and a Random Forest classifier. We picked boosting in order to compare with the boosting results in (Blasi et al., 2016), and Random Forests as a second approach often considered best-in-class. Briefly, boosting produces a prediction model in the form of an ensemble of weak prediction models, typically decision trees. The main idea behind boosting is that a set of weak learners (“specialists for specific prediction tasks”) can form a single strong learner. The name GradientBoosting reflects that a gradient descent algorithm is used to minimize a cost function when constructing the set of learners. Boosting, however, is very sensitive to mis-labeling and noise; we therefore used an additional method, Random Forests, which is also based on decision trees but typically more robust. Random Forests reduces the variance of an ensemble of “complex” models, whereas in Boosting the composition elements are “weak” models.

## 3 Results and discussion

**Fig. 5:**
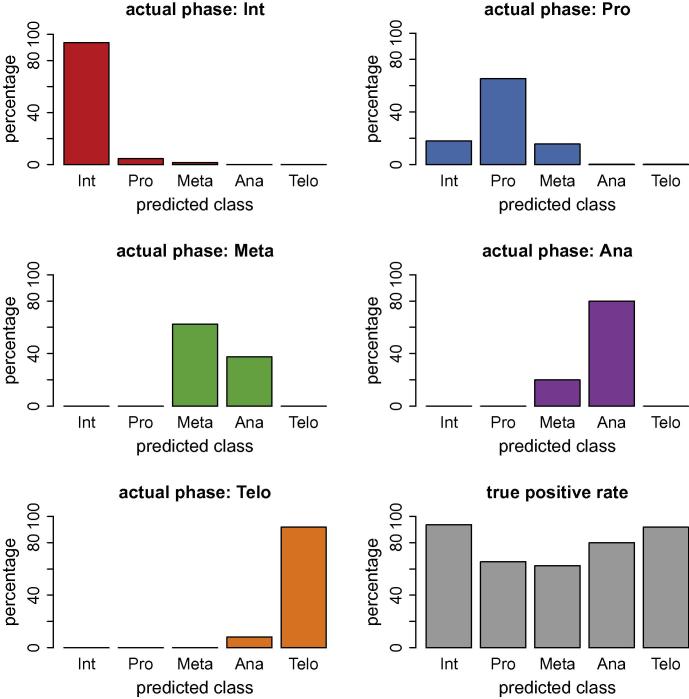
Label-free prediction of cell-cycle phases using Gradient Boosting classification. The true positive rate (which is the ratio between correctly scored phase and total number of cells in that phase) is more accurate for GradientBoosting than for Random Forests classification, in particular for metaphase and anaphase.

**Fig. 6:**
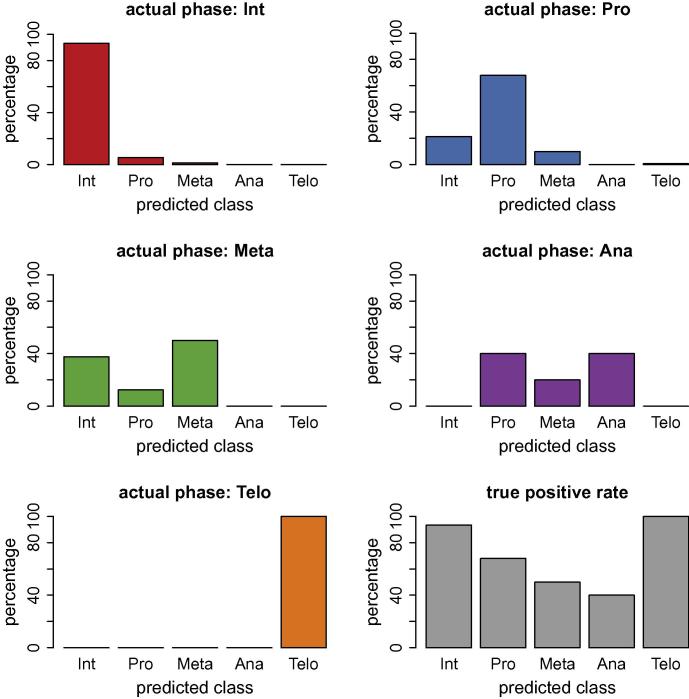
Label-free prediction of cell-cycle phases using a Random Forest classifier.

The predictions of the different cell cycle phases using our new workflow are shown in Fig. 5 for GradientBoosting and Fig. 6 for Random Forest classification. Both machine learning techniques enable a label-free classification of the cell cycle phases. GradientBoosting gave better predictions than Random Forests in our data set. Compared to Blasi et al. (2016) where a different training set and a different machine learning algorithm was used, our results are overall qualitatively similar. Interphase and telophase have high true positive rate, while prophase and metaphase are more difficult for the classifier to score correctly. The true positive rate is the number of correctly predicted cell cycle phases divided by the total number of cells presented to the trained classifier. The test set consists of over 30,000 cells, which are all cells that are not in the training set. In addition, we cross-validated the training set (10-fold cross-validation) with the evaluate function in CellProfiler Analyst.

The true positive rate for GradientBoosting in anaphase is lower compared to the prior method in Blasi et al. (2016), where a different classifier, Random Undersampling (RUS) boosting was applied. RUS boosting is tailored for highly imbalanced data sets, which may explain the superior prediction in the underrepresented class anaphase; RUS boosting is not currently an option within CellProfiler Analyst. The data set we analysed is highly imbalanced containing over 30,000 interphase cells and only 15 anaphase cells and 25 telophase cells. The relative number of cells in each phase reflects the duration of the respective cell cycle phase, i.e., the relative numbers of cells would suggest that the duration of anaphase is ∼2000 times shorter than interphase, and so on. It is crucial to compensate for this strong class imbalance. Our training set contains a lower number of cells from overrepresented classes (with respect to the total cell count), thus reducing the imbalance in the training set through undersampling. GradientBoosting detects the very few cells in the underrepresented classes with 80% (anaphase) and 92% accuracy (telophase) in the whole data set of over 30,000 cells. Fig. 5 is a graphical representation of the confusion matrices, for completeness, we provide the confusion matrices in Table 1.

**Fig. 7:**
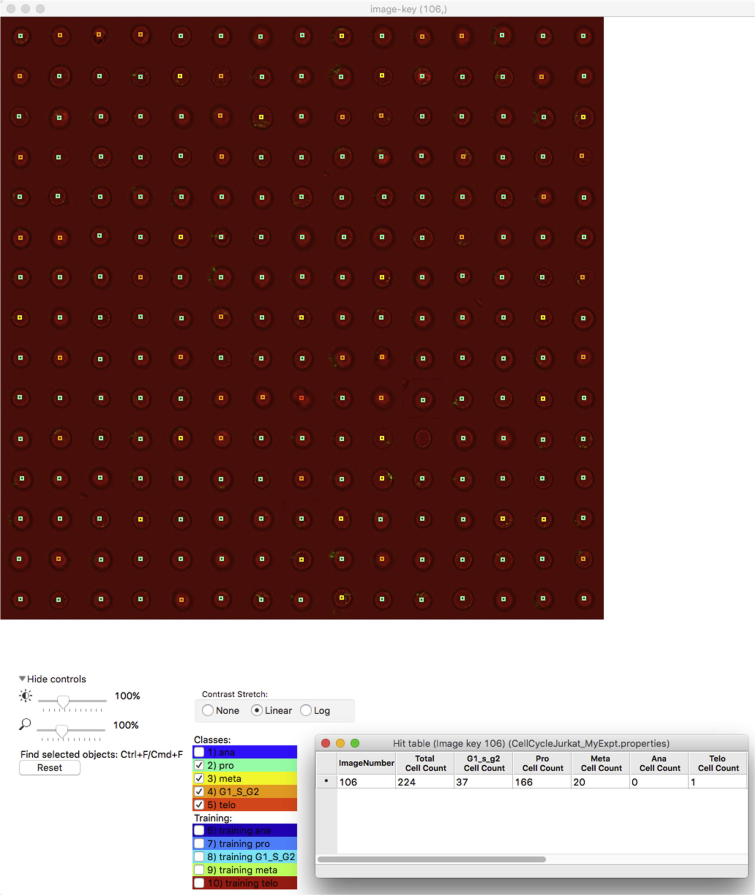
Example images of 224 cells from the test set where the cell cycle phase was predicted using machine learning (GradientBoosting). All cells displayed were deemed prophase based on our ground truth. Ground truth was obtained from the fluorescence markers via gating in IDEAS.

It can be informative to look at the images of cells in a given category. In Fig. 7, we show examples of images of 224 cells, which according to ground truth are all in prophase. We chose prophase cells for this illustration because prophase is among the most difficult classes to predict. Fig. 7 is a screenshot from CellProfiler Analyst showing the cellular images with a coloured square on top of each cell in the test set. The colour of the square denotes the predicted class, i.e., cells marked with a green square were correctly classified as prophase cells (166 out of 224 cells, cf. hit table in the lower right of the figure), whereas a yellow square marks cells that were predicted to be in metaphase (20 out of 224 cells), thus deviating from ground truth. One cell was classified as telophase (the corresponding image tile is in row 10, column 8 of the montage). Interestingly, it appears that ground truth is not correct in this case and that indeed this particular cell is not in prophase.

### 3.1 Feature selection

Which features were most informative for the prediction of the cell cycle phases? We extended our previous work [6] by identifying the most informative features. Both machine learning methods, GradientBoosting and Random Forests, include feature selection, and the top features are displayed in CellProfiler Analyst. The top 20 features are shown in Table 2 (feature #1 is the most informative, followed by feature #2 etc.).

Features extracted from the dark-field, also referred to as side scatter channel (SSC) are named SSC_∗ and bright-field features are named BF_∗. We note that the top feature for GradientBoosting is a dark-field feature, while Random Forests does not use any information from the dark-field channel among the top 20 features; instead the top 20 features stem from bright-field (BF).

## 4 Conclusions

In conclusion, we introduced an open-source and user-friendly protocol to analyse IFC data in high-throughput using machine learning based on a previously developed prototype workflow [6]. In order to demonstrate the individual steps of the new protocol, we applied machine learning to accurately predict the cell cycle phase of Jurkat cells without the use of any labels, achieving a level of accuracy comparable to the original, more cumbersome procedure. Feature selection (e.g., provided in CellProfiler Analyst) shows that the dark-field images carry valuable information for the prediction of the cell cycle phase.

Image-based flow cytometry is much more parameter-rich compared to conventional cytometry and mass cytometry approaches. Data analysis methods for IFC have fallen short of their potential. The protocol presented in this work connects imaging flow cytometers and IDEAS with powerful, high-content analyses via machine learning.

The protocol we describe will also facilitate the use of IFC data for the emerging applications using image-based profiling [25], [26]. In image-based profiling, based on hundreds of features per cell, high-content profiles are extracted and subjected to machine learning to enable new biologically relevant discoveries. We hope the open-source and user-friendly protocol contributes to IFC being more widely adopted by the research community and in clinical diagnostics. Moving forward, the development and growth of new imaging cytometry technologies, such as the CHIP Cytometer by ZellKraftwerk and Imaging Mass Cytometry are likely to be accompanied by even greater challenges posed to high-throughput image data analysis.

## Figures and Tables

**Table 1:** Confusion matrices for GradientBoosting and Random Forests classifier.

GradientBoosting		Predicted class				
		Int	Pro	Meta	Ana	Telo
True class	Int	93.63	4.64	1.63	0.07	0.03
	Pro	18.16	65.53	15.79	0.26	0.26
	Meta	0.00	0.00	62.50	37.50	0.00
	Ana	0.00	0.00	20.00	80.00	0.00
	Telo	0.00	0.00	0.00	8.33	91.67

Random Forests		Predicted class				

Inter	Pro	Meta	Ana	Telo

True class	Int	93.19	5.43	1.35	0.00	0.03
	Pro	21.32	67.89	10.00	0.00	0.79
	Meta	37.50	12.50	50.00	0.00	0.00
	Ana	0.00	40.00	20.00	40.00	0.00
	Telo	0.00	0.00	0.00	0.00	100.00

**Table 2:** The top 20 features for both the GradientBoosting and Random Forest algorithms.

*GradientBoosting: Top 20 features*	
1. SSC_Granularity_1_DF_image	11. BF_AreaShape_Zernike_2_2
2. BF_AreaShape_MajorAxisLength	12. BF_Texture_InfoMeas2_BF_image_3_0
3. BF_AreaShape_Compactness	13. BF_Intensity_MinIntensityEdge_BF_image
4. BF_AreaShape_MaxFeretDiameter	14. BF_RadialDistribution_RadialCV_BF_image_2of4
5. BF_Intensity_MeanIntensity_BF_image	15. BF_Intensity_IntegratedIntensityEdge_BF_image
6. BF_Intensity_MeanIntensityEdge_BF_image	16. BF_Intensity_LowerQuartileIntensity_BF_image
7. BF_AreaShape_MaximumRadius	17. BF_Granularity_1_BF_image
8. BF_RadialDistribution_MeanFrac_BF_image_4of4	18. BF_RadialDistribution_MeanFrac_BF_image_3of4
9. BF_AreaShape_Eccentricity	19. SSC_RadialDistribution_RadialCV_DF_image_2of4
10. BF_Intensity_MassDisplacement_BF_image	20. BF_RadialDistribution_MeanFrac_BF_image_2of4

*Random Forests: Top 20 features*	
1. BF_Intensity_IntegratedIntensityEdge_BF_image	11. BF_RadialDistribution_MeanFrac_BF_image_3of4
2. BF_Intensity_MeanIntensityEdge_BF_image	12. BF_AreaShape_Perimeter
3. BF_AreaShape_MeanRadius	13. BF_RadialDistribution_FracAtD_BF_image_2of4
4. BF_Intensity_MeanIntensity_BF_image	14. BF_AreaShape_MaxFeretDiameter
5. BF_AreaShape_MaximumRadius	15. BF_RadialDistribution_MeanFrac_BF_image_2of4
6. BF_Intensity_IntegratedIntensity_BF_image	16. BF_Intensity_MassDisplacement_BF_image
7. BF_AreaShape_MajorAxisLength	17. BF_RadialDistribution_FracAtD_BF_image_4of4
8. BF_AreaShape_MinorAxisLength	18. BF_AreaShape_Zernike_0_0
9. BF_AreaShape_Area	19. BF_AreaShape_Eccentricity
10. BF_Intensity_MinIntensityEdge_BF_image	20. BF_RadialDistribution_MeanFrac_BF_image_4of4
